# Measurement of Urinary Biomarkers of Parabens, Benzophenone-3, and Phthalates in a Belgian Population

**DOI:** 10.1155/2014/649314

**Published:** 2014-02-25

**Authors:** Lucas Dewalque, Catherine Pirard, Corinne Charlier

**Affiliations:** ^1^Laboratory of Clinical, Forensic and Environmental Toxicology, University of Liege (ULg), CHU (B35), 4000 Liege, Belgium; ^2^Center for Interdisciplinary Research on Medicines (CIRM), University of Liege (ULg), CHU (B35), | 4000 Liege, Belgium

## Abstract

Parabens, benzophenone-3 (BP3), and phthalates are commonly used as antimicrobial conservator, UV-filter, and plasticizer, respectively, and are thought to exhibit endocrine disrupting properties. These endocrine disrupting activities have been recently assumed to lead to cutaneous malignant melanoma. Humans are exposed to these chemicals through different sources such as food, personal care products, or cosmetics. In this study, we measured urinary levels of 4 parabens, BP3, and 7 metabolites of phthalates in samples collected from 261 participants living in and around Liege (Belgium). The analyses were carried out by liquid chromatography tandem mass spectrometry (LC-MS/MS) using isotopic dilution. To the best of our knowledge, this is the first time that the urinary levels of these 3 classes of chemicals are reported for the same general population in Belgium. Most of the parabens, the BP3, and all the phthalate metabolites were detected in 82.8 to 100.0% of the samples. For most of these chemicals, the exposure patterns significantly differ not only between children and adults, but also between males and females, especially with higher concentrations of parabens and phthalate metabolites in female and children subjects, respectively.

## 1. Introduction

Numerous studies have demonstrated the alarming increase of cutaneous malignant melanomas (CMM) in Caucasian populations these last decades [1–5]. CMM is known to occur mainly in women aged between 15 and 34, although the higher incidence for this specific subpopulation is not well understood [1–3]. While genetic predispositions [4] or environmental factors such as natural or artificial ultraviolet light exposure could induce CMM [5], the exposure to manmade chemicals such as persistent organic pollutants or pesticides was suspected to explain the overall increasing CMM incidence [6–8] but strong evidence is still lacking. Focusing on environmental pollutants, the endocrine disrupting chemicals, which are known to interact with the hormonal homeostasis, are thought to act on estrogen receptor present in melanoma cells [9, 10] or alter HOX genes function which seem to be correlated with tumor progression [11, 12]. Furthermore, some authors recently hypothesized a link between the higher exposure to some endocrine disrupting chemicals, namely, UV-filters and parabens, and the increasing incidence of CMM [10, 13]. In this paper, we tried to assess the human exposure of 3 classes of endocrine disruptors, namely, parabens, benzophenone-3 (BP3), and phthalates. For this purpose, we measured their urinary biomarkers.

Methyl- (MP), ethyl- (EP), n-propyl- (PP), and n-butyl-paraben (BP), which are some esters of the parahydroxybenzoic acid (PHBA), are widely used alone or in combination as an antimicrobial conservator in personal care products (cosmetics, shampoos, shaving products, lotions, etc.) but also in food, beverages, food packaging, and pharmaceutical preparations [14, 15]. When present in food, the parabens are orally absorbed and rapidly degraded by liver esterases to PHBA, which is rapidly eliminated in urine as unspecific biomarker [14]. After dermal application of personal care products containing parabens, most of them are degraded by some skin esterases and only a small fraction is available to cross the epidermis and reach the systemic circulation. The unchanged parabens are then excreted in urine as glucuronide, glycine, and sulfate conjugates and could be therefore used as specific biomarkers to assess their exposure [14, 16, 17]. Although they used to be considered as slightly toxic, the parabens have been demonstrated to show *in vitro* and *in vivo* weak estrogenic activity [18–20]. They can also alter the reproductive functions in male rats and mice after *in utero* exposure [21–23]. The human health effects of the paraben exposure at environmental levels are still unknown and their toxicity remains controversial since several studies did not achieve to demonstrate the endocrine disruptor effects [15, 24, 25]. Nevertheless, parabens have been suspected to be involved in melanocytic lesions [13] because, on the one hand, they can interact with the estrogen receptor beta [26, 27] present in melanoma cells and therefore influence the development of the tumors [9], and on the other hand, they can potentiate UV-induced damage in keratinocyte through oxidative stress [28]. It has been shown that women used to be more exposed to parabens because of their more frequent use of personal care products [29, 30]. Moreover a higher incidence of CMM has been demonstrated in women [1–3]. Consequently, the potential involvement of parabens exposure in CMM incidence can be explored, although, until now, the influence of these endocrine disruptors on the physiopathology of melanoma has never been demonstrated.

BP3 used to be added in sunscreens and cosmetics as a UV-filter but was also introduced in plastic surface coatings and polymers as a UV-stabilizer [10, 31, 32]. Following dermal exposure, BP3 is absorbed through the skin [32] and eliminated in the urine mainly as glucuroconjugated species after phase I and phase II metabolism [32, 33]. Since glucuroconjugated forms are excreted in urine in large amount, unchanged BP3 used to be monitored after hydrolysis step as a specific biomarker [32, 33]. BP3 is known to exhibit estrogen agonist properties and androgen antagonist activities [31, 34, 35]. In biomonitoring studies, higher BP3 exposure has been observed in the female population, probably also due to its presence in personal care products [36].

Phthalates are commonly used as plasticizer especially in PVC but also as solubilizing and stabilizing agent in a broad range of other applications. They can be found in various everyday life products like children toys, cosmetics, and perfumes, as well as in building materials such as vinyl flooring, in food packaging, in adhesives, in clothes, or in medical materials and drugs [57]. Since phthalates are not chemically bound to the polymers, they can be released into the environment. Their exposure can therefore occur through various sources, mainly food but also through air dust, water, use of personal care products, or parenteral way for individuals undergoing medical procedures [61]. In some animal toxicity studies, phthalates were shown to influence the endogenous production of several hormones like testosterone, insulin-like factor 3, and follicle-stimulating hormone and thus could be related to functional and structural impairment of male reproduction and development [61]. The human exposure to phthalates has been associated with alteration of sperm quality [62], reduced anogenital distance in infant [63], neurodevelopment disorders [64], and increased waist circumference and insulin resistance [65]. The exposure assessment of phthalates is carried out using biomonitoring approaches consisting in the measurement of their urinary metabolites, which are the corresponding monoesters oxidized or not [61].

This work is the first part of a larger study which will focus on the potential link between melanoma and exposure to endocrine disrupting chemicals. For this purpose, the establishment of some reference values in the Belgian general population is needed. Therefore, in order to determine these levels of background contamination, we measured urinary levels of 4 parabens (methyl-, ethyl-, propyl-, and butylparaben), BP3, and 7 metabolites of phthalates, namely, monoethyl phthalate (MEP), mono-n-butyl phthalate (MnBP), mono-iso-butyl phthalate (MiBP), monobenzyl phthalate (MBzP), mono-2-ethylhexyl phthalate (MEHP), mono-2-ethyl-5-hydroxyhexyl phthalate (5-OH-MEHP), and mono-2-ethyl-5-oxohexyl phthalate (5-oxo-MEHP), in 261 people aged between 1 and 85, living in Liege or the surrounding areas.

## 2. Material and Methods

### 2.1. Sample Collection

This study was approved by the Hospital Faculty Ethics Committee of the University of Liege (Belgium). 261 healthy females and males aged from 1 to 85, living in Liege or in the surrounding areas and having no occupational activity related to phthalates, parabens, or BP3, signed free and informed consent. The participants filled in a short questionnaire including data about age, weight, size, smoking habits, and residence localization. For children, the consent and the questionnaire were filled in by the parents or the person in charge. The characteristics of the study population are detailed in Table 1. As summarized in this table, the participants were classified into 3 groups depending on their residence place and based on the Eurostat concept of the rural and urban communities [66]. Therefore these places of residence were defined according to the population density and the total number of inhabitants as densely populated (>500 inhabitants/km² and ≥50,000 inhabitants), intermediately populated (between 100 and 500 inhabitants/km² and ≥50,000 inhabitants), and sparsely populated (≤100 inhabitants/km² and <50,000 inhabitants). Spot urine samples were collected in 100 mL polypropylene containers previously screened for potential contamination of phthalate metabolites, BP3, and parabens. The sample collection was carried out from January to April 2013. Immediately after the collection, samples were aliquoted and frozen at −20°C since the phthalate metabolites were demonstrated to be stable in these conditions for at least one year [67] and parabens and BP3 for 6 months [68].

### 2.2. Phthalate Metabolites, Parabens, and BP3 Analysis

The optimization and validation of the analytical procedure for the simultaneous determination of the 7 phthalate metabolites, the 4 parabens, and the BP3 have been previously described [69]. Briefly, after the addition of internal standard and sodium acetate buffer to 3 mL of previously centrifuged urine, the samples were hydrolyzed overnight at 37°C using *Helix pomatia* glucuronidase. Then samples were acidified using 200 *μ*L of formic acid, centrifuged again, and the supernatants were loaded on the SPE Bond Elut Certify LRC cartridges which had previously been conditioned. The cartridges were then washed with acetic acid and eluted twice with acetonitrile. The eluate was then evaporated until dryness under a nitrogen gentle flow at 40°C and reconstituted in 70 *μ*L of a 70 : 30 (v:v) acidified water-acetonitrile solution. Finally, the extracts were centrifuged one last time prior to analysis, performed by UHPLC-MS/MS in positive electrospray mode (ESI) for BP3 and negative ESI for parabens and phthalate metabolites. The separation was carried out using a Kinetex Phenyl-Hexyl column 100 × 2.1 mm, 1.7 *μ*m with acidified water and acetonitrile as mobile phases. The LC gradient, the specific parameters of the mass spectrometry, and the characteristics of the MS/MS transitions have been detailed elsewhere [69].

### 2.3. Urinary Creatinine Determination

The creatinine measurements were carried out using the automate ARCHITECT ci 4100 (Abbott, Illinois, USA) and the Abbott reagents and calibration kits. The analysis method was based on enzymatic chain reactions and absorbance measurements.

### 2.4. Determination of Unknown Samples

The determination of unknown samples was carried out using calibration curves ranging from 0.5 to 200 *μ*g/L (except MP and BP3 from 2 to 800 *μ*g/L) in synthetic urine. When the concentration measured was above the highest calibration point, the analysis was rerun on diluted samples with synthetic urine. Each sequence of unknown samples included a procedural blank (constituted of synthetic urine) and two level home-made quality controls (10 and 100 *μ*g/L for each compound except BP3 and MP, 40, and 400 *μ*g/L) [69]. Moreover, our lab participated and successfully passed the German External Quality Assessment Scheme (G-EQUAS) 2013 program, in which human urine control materials 51-9A and 51-9B were analyzed for MnBP, MiBP, MBzP, MEHP, 5-OH-MEHP, and 5-oxo-MEHP [69].

### 2.5. Statistical Analysis

The values below our limits of detection (LOD) were treated as LOD/2 in the statistical analyses [37, 43, 47, 70]. Kruskal-Wallis test, Mann-Whitney *U* test and Spearman's rank correlation were performed using GraphPad Prism 5.0 software (GraphPad Software, CA, USA) to compare biomarker levels measured according to the age group and the gender and to highlight associations. Microsoft Office Excel 2003 (Microsoft Corporation, Washington, USA) was used to determine percentiles and geometric means (GM). Significance limit was set at 0.05.

## 3. Results and Discussion

Creatinine adjustment is commonly used to take into account the volume dilution in environmental biomonitoring studies. Actually several studies suggested that creatinine adjustment could induce bias when comparing different populations such as ethnical groups, pregnant women, neonatal, children, or the elderly for whom creatinine excretion could be impacted by physiological factor not directly related to their environmental exposure, for instance, renal function, muscle mass, sex, ethnicity, food consumption, and age [57, 59, 71–75]. For these reasons, creatinine adjustment is more and more discouraged in biomonitoring studies [76]. Therefore, the results are presented here in both *μ*g/L and *μ*g/g creatinine, but all statistical analyses and discussions were performed on unadjusted concentrations. For each of biomarkers measured, unadjusted urinary levels were highly or very highly correlated with their respective creatinine adjusted concentrations (*r* = 0.75–0.97 *P* < 0.001) excepted for MEHP for which correlation was moderate (*r* = 0.56  *P* < 0.001). The Mann-Whitney *U* test did not highlight any significant difference in biomarkers levels according to the place of residence. No statistics were performed on the influence of smoking habits because of the very small proportion of smokers in the studied population (Table 1).

GM, the percentiles (5th, 25th, 50th, 75th, and 95th), the range, and the frequencies of detection are detailed in Table 2 for the 261 participants. All subjects were categorized into six age groups (1–6; 7–11; 12–19; 20–39; 40–59; ≥60 years) including a minimum of 23 participants and homogeneously distributed according to the sex (Table 1). The median biomarker levels were also presented according to the different age groups in Table 3.

### 3.1. Parabens

MP was detected in all the urine samples at concentrations ranging from 0.3 to 7576 *μ*g/L and at a GM of 19.0 *μ*g/L (Table 2). EP and PP were positively detected in 96.6% and 83.1% of the urine samples, respectively, and their GM levels were 2.1 *μ*g/L and 1.5 *μ*g/L, ranging from <LOD to 887 *μ*g/L and <LOD to 692 *μ*g/L, respectively. Unlike the other parabens, BP showed a poor detection rate (41%) which did not allow us to determine GM and perform statistics and showed globally lower urinary levels (from <LOD to 81 *μ*g/L). Whatever the targeted paraben is, significantly higher levels were observed in the urine of women compared to men (*P* = 0.040–<0.0001). This observation was consistent with the NHANES study on the American general population [29] and was most likely related to the higher use by women of personal care products such as cosmetics which may contain parabens. Moreover, a recent study highlighted the association between fresh application of cosmetics and higher paraben exposure [30]. Focusing on the urinary paraben levels according to the age group (Table 3), EP levels were significantly lower in the age group of 7–11 years, while conversely BP concentrations were statistically higher in young children (1–6 years) compared to teenagers and young adults (12–39 years) and to the older group (≥60 years). If higher EP levels in the adults could be probably explained by more important use of personal care products or pharmaceutical preparations containing EP, the reason why young children seemed to be more exposed to EP and BP was unclear. On the other hand, the levels of the four studied parabens were correlated (*r* = 0.46–0.79 *P* < 0.001) and especially MP and PP (*r* = 0.79  *P* < 0.001) as detailed in Table 4. This suggested potential common sources of exposure for the different parabens known to be used in combination in personal care products, pharmaceutical preparations, or food [15, 16]. Furthermore, MP and PP are reported to be more frequently combined parabens [16] and were also strongly correlated in other biomonitoring studies [29, 30, 37, 39, 41]. Conversely, Shirai et al. [43] did not observe such a significant correlation between parabens in Japanese pregnant women. The apparent inconsistence with the Asian study might be the result of different paraben use in commercial products from one country to another, yielding to different exposure between populations.

Table 5 gathers the urinary paraben results from different national large-scale biomonitoring studies for children and adults. The highest paraben concentrations in children urine were reported in four-year-old Spanish boys [37]. Excluding this Spanish study, the paraben levels found in our Belgian children seemed to be close to those usually measured in other countries except for MP detected, respectively, at higher and lower urinary concentrations than in some Danish and American children [38, 39]. On the other hand, the Belgian men seemed to be less exposed to all parabens than the Danish or American male populations [38, 41]. Focusing on women results, more data used to be available on urinary paraben levels and more specifically for pregnant women. In the present study, the levels measured in the women urine were overall quite lower than those reported in French, Spanish, Japanese, American, or Puerto Rican women but slightly higher than those described in some Danish mothers [30, 37–39, 42, 43]. It is of note that the lower BP levels and detection rate were also observed in all biomonitoring surveys, and even if the use of specific paraben according to the application could be variable between countries, the profiles were consistent in all studies with MP sharing for 75 to 90%, followed by PP and EP.

### 3.2. BP3

BP3 was detected in 82.8% of analyzed samples with levels ranging from <LOD to 662.8 *μ*g/L and a GM level of 1.3 *μ*g/L (Table 2). Similar to parabens, since BP3 used to be frequently incorporated in personal care products, its urinary levels were demonstrated to be correlated with the use of cosmetics [30]. Nevertheless, no significant difference was observed between males and females in the present study (*P* = 0.086) unlike in the NHANES study [36]. BP3 levels measured were significantly higher in adolescents (12–19 years) compared to adults (Table 3). This higher exposure for adolescents could not be reasonably explained. Besides the slight but significant correlation between BP3 and parabens (*r* = 0.27–0.37 *P* < 0.001) already observed in a previous study [30], BP3 seemed to be weakly correlated with some phthalate metabolites, mainly MnBP, MiBP, 5-OH-MEHP, and 5-oxo-MEHP (*r* = 0.33–0.37 *P* < 0.001). Although personal care products are known to be a source of exposure for both BP3 and parabens, other BP3 exposure routes have been suggested such as sunscreens or plastic surface coatings for food packaging [10, 31, 36]. We suspected this plastic food packaging to be a common route of exposure for BP3 and phthalates, therefore explaining the correlation found between both chemicals classes. On the other hand, the phthalates and BP3 are used in a wide range of other applications in the everyday life, and therefore a weak correlation was not unexpected [10, 61].

Compared to other surveys (Table 5), BP3 levels measured in the present study were fairly similar to those observed in child or adult population from different countries [37, 42] except in USA or in Puerto Rico [30, 36, 39] where levels found were up to 10- to 20-fold higher. This higher exposure would most likely be the reflection of the higher use of BP3 in North America where, for instance, 59% of the sunscreens were reported to contain this chemical [77]. Conversely, the urinary BP3 levels observed in China were much lower than those measured in Belgium [40, 44].

### 3.3. Phthalate Metabolites

**Figure 1:**
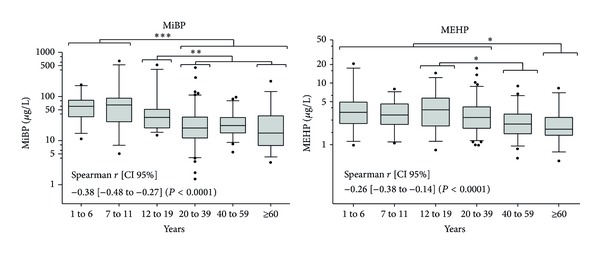
Urinary concentrations of MiBP and MEHP [*μ*g/L] according to the age groups. The lower and upper boundaries of the boxes represent the 25th and 75th percentile, respectively. The line within the box is the median level and the whiskers are the 5th and 95th percentiles. Spearman's rank correlations [95% confidence interval] between metabolite concentrations and age are mentioned. *P < 0.05; **P < 0.01; ***P < 0.001.

The phthalate metabolites were positively detected in nearly all urine samples analyzed (Table 2). The GM ranged from 2.7 to 8.6 *μ*g/L for MBzP, MEHP, 5-oxo-MEHP, and 5-OH-MEHP while higher levels were observed for MEP, MnBP, and MiBP with GM ranging between 26.2 and 37.6 *μ*g/L. We did not observe any significant difference in urinary phthalate metabolite concentrations between males and females except for the sum of the metabolites of the diethylhexylphthalate (MEHP, 5-oxo- MEHP, and 5-OH-MEHP) which was statistically higher in men (*P* = 0.0166). The distribution of the metabolite levels is presented according to the age classification for MiBP and MEHP as an example in Figure 1. This figure details the significant differences which exist between the different age groups for both metabolites, while the global significant observations are shown in Table 3. As it was previously reported [47, 49, 78], the levels observed in children were quite higher than in adults. This reinforces the assumption raised by Silva et al. [78] about higher phthalate exposure for children relating to more time spent indoors and therefore the higher exposure to the phthalates potentially found in the household environment such as in carpets, vinyl flooring, pigments, or paints [79, 80]. Furthermore, children are known to have higher respiratory rates leading to higher exposure through indoor air and house dust [81, 82]. Their relatively higher food intake/body-weight ratio could also result in higher exposure than adults [78].

Some moderate to very high correlations were observed among the phthalate metabolites (Table 4). As expected, the three metabolites of diethylhexylphthalate (DEHP) were highly correlated (*r* = 0.69–0.96 *P* < 0.001). A stronger association between both oxidized metabolites of DEHP was observed compared to the correlation between oxidized metabolites and MEHP. This is consistent with some previous studies [83, 84], and one of the reasons for this lower correlation rate might be explained by the differences in half-time elimination between oxidized DEHP metabolites and MEHP [61]. MiBP, MnBP, and MBzP were moderately to highly correlated (*r* = 0.60–0.68 *P* < 0.001) but also with 5-oxo-MEHP and 5-OH-MEHP (*r* = 0.54–0.62 *P* < 0.001). MEP was moderately associated with MnBP and MBzP (*r* = 0.51 and 0.44 <0.001, resp.) but weakly with other phthalate metabolites (*r* = 0.18–0.32 *P* < 0.001). These results suggest that Belgians seemed to be exposed to some mixtures of phthalates through similar routes. Göen et al. [83] and Frederiksen et al. [85] also reported roughly comparable correlations between phthalate metabolites, but some correlation rates could slightly differ illustrating the variability of the exposure pattern of phthalates through the European countries.

During the past decade, numerous biomonitoring studies focused on the phthalate metabolites in the general population or in some specific subpopulations. A nonexhaustive comparison between different national large-scale studies in children and adults is presented in Table 6. The levels of the phthalate metabolites measured in the present study were fairly similar to those observed in the Belgian children and mothers recruited during the recent DEMOCOPHES study [49].

Focusing on the child population, the sum of the phthalate metabolites in the Belgian urine samples was comparable to those reported from Denmark [39], Taiwan [45], and Canada [47] but higher than in the CDC study [38] and lower in German [46] and Korean children [48]. Except in Spain where the highest phthalate metabolite levels were measured [37] either for children or adults, the levels measured in our adult participants seemed to be close to those reported in most of the other adult populations from Europe, Asia, or North America [38, 44, 47, 50, 54, 59, 60]. Nevertheless, quite higher urinary levels were reported in some studies such as in France, The Netherlands, Germany, or Mexico [51, 53, 56, 57] while very low urinary concentrations were measured in pregnant Peruvian women [52]. The urine of the present Belgian children and adults seemed to show a different phthalate metabolite profile, characterized by higher proportions of MnBP and MiBP compared to MEP for children and a higher MEP excretion rate for adults. This different profile could be related to a different exposure pattern for children and adults, with a higher exposure to diethyl phthalate due to higher use of personal care products by adults [86] compared to children. For the latter, the potential contamination of the interior environment could be considered as an important pathway of exposure [78]. These exposure patterns observed could be country dependent and probably related to different food or lifestyle habits and specific commercial use of phthalates. For example, the Chinese adults [59] showed higher MiBP and MnBP levels than MEP while the French or Mexican women [56, 57] presented a greater level of the metabolites of DEHP compared to other phthalate biomarkers.

## 4. Conclusion

This study reported for the first time, to the best of our knowledge, the simultaneous measurement of 7 phthalate metabolites, 4 parabens, and the BP3 in 261 participants from the Belgian general population aged from 1 to 85 years. Although this work presents several limitations in terms of representativeness such as a low sample number, limited sampling localization, and a small socioeconomic diversity, our results were close to the Belgian DEMOCOPHES references values [49]. As reported in other biomonitoring studies, we observed widely spread population exposure to these endocrine disruptor chemicals. The urinary paraben levels observed in the present study were statistically higher in women. Because the skin effects of alkyl parabens at environmental doses are still unknown, their potential interaction with CMM cells should be investigated considering that exposure for the women seemed to be higher due to the use of personal care products. EP, BP3, and phthalate metabolites (excepted MEP) showed significant different urinary levels according to the age groups. Higher exposure in younger age groups is a matter of concern since the disruption of hormonal balance during the development stage might have long-term consequences on their health. The results obtained in this study showed some important differences in terms of exposure levels and pattern among different countries but also among participants in the same population. The sum of the twelve targeted compounds ranged between 14.8 and 8575.2 *μ*g/L showing that the cumulative exposure might be 600 times higher from one individual to another. This is also a matter of concern since additive endocrine disrupting effects are to be expected [87].

## Figures and Tables

**Table 1:** Demographic details on the studied population.

	Men	Women
*N* (%)	123 (47.1%)	138 (52.9%)
1 to 6 years	12	11
* *1 to 3 years	*3 *	*6 *
* *4 to 6 years	*9 *	*5 *
7 to 11 years	11	14
12 to 19 years	15	15
20 to 39 years	46	53
40 to 59 years	24	26
≥60 years	15	19
Average age (min–max) (years)	31.3 (2–75)	31.9 (1–85)
BMI (kg/m²)		
BMI < 18.5	23.5%	23.9%
18.5 ≤ BMI < 25	60.9%	46.0%
25 ≤ BMI < 30	11.7%	23.9%
BMI ≥ 30	3.9%	6.2%
Placed residence		
Densely populated	51.3%	51.1%
Intermediately populated	43.5%	40.6%
Sparsely populated	5.2%	8.3%
Smoker		
Yes/no	6.1%/93.9%	5.4%/94.6%

**Table 2:** Urinary concentrations of parabens, BP3, and phthalate metabolites (µg/L or µg/g creatinine): geometric means (GM), percentiles, ranges, and positivity rates.

	GM µg/L (µg/g creat.)	Selected percentiles µg/L (µg/g creat.)					Range µg/L (µg/g creat.)	Positive samples (%)^a^	LOD (µg/L)
		5th	25th	50th	75th	95th			
MP									
All (*n* = 261)	**19.0 (16.9)**	**1.1 (1.0)**	**4.3 (3.6)**	**16.1 (14.3)**	**75.2 (61.1)**	**462.6 (501.7)**	**0.30–7576.0 (0.23–3712.0)**	**100.0**	0.16
Male (*n* = 123)	10.1 (7.8)	1.0 (1.0)	3.3 (2.8)	7.7 (5.1)	26.9 (16.4)	223.4 (263.5)	0.30–2659.0 (0.23–2227.0)	100.0	
Female (*n* = 138)	33.5 (33.5)***	1.0 (1.5)	11.0 (9.8)	32.4 (32.1)	115.1 (128.7)	630.6 (612.7)	0.37–7576.0 (0.28–3712.0)	100.0	
EP									
All (*n* = 261)	**2.1 (1.8)**	**0.1 (0.2)**	**0.6 (0.5)**	**1.7 (1.5)**	**6.5 (5.7)**	**67.7 (53.9)**	**<LOD–887.3 (<LOD–1033.0)**	**96.6**	0.09
Male (*n* = 123)	1.6 (1.3)	0.1 (0.1)	0.5 (0.4)	1.3 (1.0)	4.7 (3.5)	41.4 (26.4)	<LOD–887.3 (<LOD–592.3)	95.9	
Female (*n* = 138)	2.6 (2.6)*	0.1 (0.2)	0.8 (0.7)	1.9 (2.1)	9.2 (8.8)	83.1 (90.5)	<LOD–452.8 (<LOD–1033.0)	97.1	
PP									
All (*n* = 261)	**1.5 (1.3)**	**<LOD (<LOD)**	**0.2 (0.2)**	**1.2 (1.0)**	**9.3 (8.4)**	**78.8 (89.2)**	**<LOD–692.1 (<LOD–415.1)**	**83.1**	0.11
Male (*n* = 123)	0.6 (0.5)	<LOD (<LOD)	0.1 (0.1)	0.5 (0.4)	2.2 (1.6)	20.2 (15.0)	<LOD–114.6 (<LOD–156.2)	76.4	
Female (*n* = 138)	3.3 (3.3)***	<LOD (<LOD)	0.8 (0.6)	3.3 (3.5)	15.4 (19.6)	116.5 (215.1)	<LOD–692.1 (<LOD–415.1)	89.1	
BP									
All (*n* = 261)	**ND (ND)**	**<LOD (<LOD)**	**<LOD (<LOD)**	**<LOD (<LOD)**	**0.9 (0.8)**	**8.0 (8.5)**	**<LOD–80.6 (<LOD–63.5)**	**41.8**	0.30
Male (*n* = 123)	ND (ND)	<LOD (<LOD)	<LOD (<LOD)	<LOD (<LOD)	0.3 (0.2)	4.9 (3.8)	<LOD–19.4 (<LOD–12.4)	23.6	
Female (*n* = 138)	ND (ND)***	<LOD (<LOD)	<LOD (<LOD)	0.5 (0.5)	1.7 (1.7)	11.1 (10.3)	<LOD–80.6 (<LOD–63.5)	58.0	
MEP									
All (*n* = 261)	**37.6 (33.3)**	**4.9 (5.7)**	**16.0 (14.7)**	**34.3 (30.6)**	**88.5 (68.4)**	**292.1 (251.8)**	**2.23–1904.0 (2.73–860.9)**	**100.0**	0.28
Male (*n* = 123)	40.2 (31.1)	5.2 (5.2)	15.6 (15.6)	34.3 (34.3)	93.9 (93.9)	731.9 (731.9)	2.65–1904.0 (2.65–1904.0)	100.0	
Female (*n* = 138)	35.4 (35.4)	4.9 (4.9)	16.5 (16.5)	34.5 (34.5)	85.2 (85.2)	216.9 (216.9)	2.23–578.0 (2.23–578.0)	100.0	
MnBP									
All (*n* = 261)	**31.3 (27.7)**	**5.7 (7.1)**	**15.5 (14.7)**	**33.3 (26.1)**	**63.1 (53.4)**	**145.9 (112.6)**	**2.00–235.6 (3.63–422.0)**	**100.0**	0.30
Male (*n* = 123)	33.0 (25.5)	6.7 (6.9)	17.0 (12.1)	34.8 (26.1)	61.1 (46.9)	150.4 (91.1)	3.23–222.7 (3.63–143.1)	100.0	
Female (*n* = 138)	29.8 (29.9)	5.3 (8.6)	14.9 (15.5)	31.4 (26.1)	63.4 (60.4)	116.5 (126.7)	2.00–235.6 (4.66–422.0)	100.0	
MiBP									
All (*n* = 261)	**26.2 (23.3)**	**5.9 (6.9)**	**14.3 (12.4)**	**24.3 (20.0)**	**45.9 (38.5)**	**154.4 (147.3)**	**2.04–608.4 (3.09–473.1)**	**100.0**	0.37
Male (*n* = 123)	24.9 (19.3)	5.7 (5.4)	13.5 (10.5)	24.3 (17.2)	43.4 (29.0)	99.6 (99.7)	2.46–504.9 (3.09–307.2)	100.0	
Female (*n* = 138)	27.5 (27.5)	6.2 (8.6)	15.0 (14.3)	24.6 (22.4)	48.1 (50.5)	169.5 (174.3)	2.04–608.4 (5.93–473.1)	100.0	
MBzP									
All (*n* = 261)	**5.5 (4.9)**	**0.9 (1.2)**	**2.9 (2.3)**	**5.5 (4.4)**	**10.3 (9.1)**	**34.9 (28.5)**	**<LOD–126.5 (<LOD–102.0)**	**99.6**	0.19
Male (*n* = 123)	5.8 (4.5)	1.0 (1.0)	3.0 (3.0)	5.8 (5.8)	11.3 (11.3)	40.5 (40.5)	0.40–126.5 (0.65–102.0)	100.0	
Female (*n* = 138)	5.2 (5.2)	0.8 (0.8)	2.6 (2.6)	5.3 (5.3)	10.1 (10.1)	34.3 (34.3)	<LOD–84.0 (<LOD–73.0)	99.3	
MEHP									
All (*n* = 261)	**2.7 (2.4)**	**1.1 (0.7)**	**1.7 (1.5)**	**2.7 (2.3)**	**4.1 (4.0)**	**8.7 (7.8)**	**0.58–20.0 (0.51–46.5)**	**100.0**	0.19
Male (*n* = 123)	3.0 (2.4)**	1.3 (0.7)	1.9 (1.5)	3.0 (2.1)	4.6 (3.6)	8.7 (6.8)	0.99–13.4 (0.51–9.7)	100.0	
Female (*n* = 138)	2.5 (2.5)	1.0 (0.7)	1.5 (1.4)	2.2 (2.3)	3.8 (4.2)	8.7 (8.5)	0.58–20.0 (0.56–46.5)	100.0	
5-OH-MEHP									
All (*n* = 261)	**8.6 (7.6)**	**1.8 (2.0)**	**4.7 (4.3)**	**9.0 (6.8)**	**15.3 (13.4)**	**36.7 (35.1)**	**0.31–113.0 (0.96–263.5)**	**100.0**	0.13
Male (*n* = 123)	10.1 (7.8)**	2.5 (2.0)	5.6 (4.5)	9.9 (7.3)	19.4 (13.8)	37.3 (33.4)	1.33–59.3 (1.47–41.8)	100.0	
Female (*n* = 138)	7.4 (7.4)	1.2 (1.8)	3.7 (4.1)	7.9 (6.5)	13.7 (12.2)	37.4 (36.4)	0.31–113.0 (0.96–263.5)	100.0	
5-oxo-MEHP									
All (*n* = 261)	**5.8 (5.1)**	**0.9 (1.4)**	**3.1 (2.8)**	**5.9 (4.8)**	**11.2 (8.7)**	**27.4 (23.4)**	**0.29–94.7 (0.67–220.7)**	**100.0**	0.16
Male (*n* = 123)	6.5 (5.0)	1.4 (1.4)	3.8 (2.7)	6.7 (4.8)	12.2 (9.0)	26.9 (21.9)	0.87–57.4 (0.67–36.8)	100.0	
Female (*n* = 138)	5.3 (5.3)	0.8 (1.4)	2.9 (2.9)	5.6 (4.9)	10.2 (8.1)	35.2 (26.6)	0.29–94.7 (0.80–220.7)	100.0	
BP3									
All (*n* = 261)	**1.3 (1.1)**	**<LOD (<LOD)**	**0.4 (0.3)**	**1.3 (1.0)**	**3.7 (3.1)**	**32.2 (30.1)**	**<LOD–662.8 (<LOD–414.2)**	**82.8**	0.20
Male (*n* = 123)	1.1 (0.8)	<LOD (<LOD)	0.3 (0.2)	0.9 (0.6)	3.1 (2.0)	34.5 (28.8)	<LOD–662.8 (<LOD–414.2)	82.1	
Female (*n* = 138)	1.4 (1.4)	<LOD (<LOD)	0.4 (0.4)	1.7 (1.3)	3.8 (4.4)	32.7 (33.3)	<LOD–140.0 (<LOD–141.3)	83.3	

^a^samples above LOD (%).

*P < 0.05; **P < 0.01; ***P < 0.001.

**Table 3:** Paraben, BP3, and phthalate metabolite medians (µg/L) in the different age groups.

Age groups (*N*)	1–6 (23)	7–11 (25)	12–19 (30)	20–39 (99)	40–59 (50)	≥60 (34)
MP	34.8	9.1	18.0	13.3	25.6	13.0
EP	2.3*	0.7	1.1	1.9*	2.2*	2.6*
PP	2.1	0.8	4.2	1.0	2.3	0.8
BP	0.8*	<LOD	<LOD	<LOD	<LOD	<LOD
BP3	1.8	1.4	3.6**	0.9	1.3	0.5
MEP	33.3	39.2	42.4	27.5	34.5	54.0
MnBP	59.0***	48.4*	40.9*	24.8	30.7	29.4
MiBP	59.5***	64.1***	33.6**	19.3	21.9	14.8
MBzP	10.2*	8.2*	8.4*	4.2	4.1	4.6
MEHP	3.4**	3.0*	3.7*	2.8*	2.2	1.8
5-OH-MEHP	21.7***	14.3**	13.7***	7.3	5.8	6.0
5-oxo-MEHP	17.1***	10.2***	9.5***	4.6	3.8	3.8

*P < 0.05; **P < 0.01; ***P < 0.001.

**Table 4:** Spearman's rank correlations between urinary phthalate metabolites, parabens, and BP3.

	MEP	MnBP	MiBP	MBzP	MEHP	5-OH-MEHP	5-oxo-MEHP	MP	EP	PP	BP	BP3
MEP	—											
MnBP	0.51***	—										
MiBP	0.32***	0.64***	—									
MBzP	0.44***	0.68***	0.60***	—								
MEHP	0.18***	0.35***	0.34***	0.42***	—							
5-OH-MEHP	0.27***	0.54***	0.56***	0.62***	0.69***	—						
5-oxo-MEHP	0.24***	0.54***	0.59***	0.60***	0.70***	0.96***	—					
MP	0.25***	0.20**	0.19**	0.14*	0.07^ns^	0.12^ns^	0.12*	—				
EP	0.35***	0.30***	0.19**	0.15*	0.04^ns^	0.14*	0.08^ns^	0.55***	—			
PP	0.25***	0.23***	0.17**	0.11^ns^	0.09^ns^	0.08^ns^	0.07^ns^	0.79***	0.48***	—		
BP	0.17**	0.35***	0.32***	0.24***	0.16**	0.24***	0.25***	0.51***	0.46***	0.53***	—	
BP3	0.25***	0.35***	0.37***	0.25***	0.15*	0.33***	0.33***	0.27***	0.28***	0.33***	0.37***	—

*P < 0.05; **P < 0.01; ***P < 0.001; ns: not significant.

**Table 5:** Paraben and BP3 concentrations—medians (95th percentile) in µg/L—reported in human urine samples for children, males, and females.

Location (sampling years)	Population	Age (years)	*N*	MP	EP	PP	BP	BP3	Reference
**Belgium (2013)**	**Children**	**1–11**	**48**	**18.6 (581.1)**	**1.1 (13.7)**	**1.1 (96.9)**	**0.5 (5.7)**	**1.6 (15.2)**	**This study**
Spain (2005-2006)	Boys	4	30	150.0 (—)	8.1 (—)	21.5 (—)	1.2 (—)	1.9 (—)	[37]
USA (2009-2010)	Children	6–11	415	26.5 (873.0)	<LOD (11.5)	2.7 (114.0)	<LOD (2.2)	14.6 (1570.0)	[38]
Denmark (2011)	Children	6–11	143	3.0 (62.0)	0.4 (3.7)	1.7 (33.0)	<LOD (1.4)	1.8 (40.0)	[39]
China (2012)	Children	9-10	70	— (—)	— (—)	— (—)	— (—)	0.6 (6.4)^‡^	[40]
**Belgium (2013)**	**Males**	**1–75**	**123**	**7.7 (223.4)**	**1.3 (41.4)**	**0.5 (20.2)**	**<LOD (4.9)**	**0.9 (34.5)**	**This study**
Denmark (2006)	Males	18–26	60	17.7 (2002.0)^‡^	2.0 (564.0)^‡^	3.6 (256.0)^‡^	0.2 (67.6)^‡^	— (—)	[41]
USA (2009-2010)	Males	≥6	1399	25.3 (727.0)	<LOD (36.4)	2.8 (134.0)	<LOD (2.7)	15.3 (610.0)	[38]
**Belgium (2013)**	**Females**	**1–85**	**138**	**32.4 (630.6)**	**1.9 (83.1)**	**3.3 (116.5)**	**0.5 (11.1)**	**1.7 (32.7)**	**This study**
France (2002–2006)	Pregnant women	—	191	104.3 (2689.7)	1.5 (38.2)	10.4 (267.7)	2.2 (63.6)	1.7 (143.0)	[42]
Spain (2004–2008)	Pregnant women	—	120	191.0 (—)	8.8 (—)	29.8 (—)	2.4 (—)	3.4 (—)	[37]
Japan (2007–2010)	Pregnant women	32.6^¥^	111	75.8 (1361.0)^‡^	7.5 (593.0)^‡^	20.2 (2690.0)^‡^	0.6 (22.8)^‡^	— (—)	[43]
USA (2009-2010)	Females	≥6	1350	106.0 (1230.0)	2.0 (138.0)	20.2 (361.0)	0.3 (31.8)	32.0 (3200.0)	[38]
Puerto Rico (2010–2012)	Pregnant women	—	105	153.0 (1590.0)	— (—)	36.7 (493.0)	0.4 (36.4)	31.3 (2150.0)	[30]
China (2010–2012)	Pregnant women	≥18	567	— (—)	— (—)	— (—)	— (—)	0.1 (0.8)	[44]
Danemark (2011)	Mothers	31–52	145	14.0 (275.0)	0.9 (44.0)	<LOD (14.0)	<LOD (9.3)	3.7 (312.0)	[39]

—: no results/information.

^‡^maximum.

^¥^mean.

**Table 6:** Phthalate metabolite concentrations—median (95th percentile) in µg/L—reported in human urine samples for children and adults.

Location (sampling years)	Population	Age (years)	*N *	MEP	MnBP	MiBP	MBzP	MEHP	5-OH-MEHP	5-oxo-MEHP	Reference
**Belgium (2013)**	**Children**	** 1–11**	**48**	**35.6 (139.6)**	**55.7 (132.7)**	**61.8 (175.8)**	**9.7 (52.8)**	**3.1 (7.2)**	**18.7 (61.7)**	**12.3 (47.0)**	**This study**
Taiwan (2001-2002)^a^	Children	2–6	89	— (—)	87.9 (16455.0)^‡^	21.9 (252.7)^‡^	3.8 (69.4)^‡^	8.1 (94.7)^‡^	39.6 (1014.0)^‡^	31.0 (761.0)^‡^	[45]
Germany (2003–2006)	Children	3–14	599	— (—)	93.4 (310.0)	88.1 (308.0)	18.1 (76.2)	6.7 (25.1)	46.0 (164.0)	36.3 (123.0)	[46]
Spain (2005-2006)	Children boys	4	30	324.0 (—)	30.2 (—)	41.9 (—)	33.0 (—)	6.2 (—)	57.4 (—)	44.6 (—)	[37]
Canada (2007–2009)	Children	6–11	1037	23.6 (210.7)	32.6 (168.2)	— (—)	21.4 (131.1)	6.4 (17.8)	31.6 (179.5)	20.3 (106.7)	[47]
USA (2009-2010)	Children	6–11	415	33.0 (288.0)	23.3 (124.0)	10.9 (55.4)	12.6 (87.8)	1.7 (8.9)	17.0 (75.1)	11.1 (48.4)	[38]
Denmark (2011)	Children	6–11	143	20.0 (68.0)	32.0 (99.0)	54.0 (193.0)	7.0 (31.0)	2.0 (10.0)	23.0 (89.0)	12.0 (40.0)	[39]
Korea (2011)	Children	0–6	392	— (—)	— (—)	— (—)	— (—)	14.9 (58.1)	80.3 (253.2)	83.3 (265.5)	[48]
Belgium (2011-2012)	Children	6–11	125	23.0 (169.0)	40.0 (122.0)	54.0 (362.0)	8.6 (27.0)	2.2 (8.7)	17.0 (31.0)	13.0 (22.0)	[49]
**Belgium (2013)**	**Males and females**	** 12–85**	**213**	**34.3 (396.3)**	**30.2 (142.2)**	**20.1 (89.3)**	**4.6 (26.5)**	**2.5 (8.7)**	**7.4 (30.6)**	**4.9 (19.1)**	**This study**
Sweden (2001)	Mothers	23–39	38	35.0 (761.0)^‡^	46.0 (198.0)^‡^	16.0 (130.0)^‡^	13.0 (38.0)^‡^	9.0 (57.0)^‡^	15.0 (126.0)^‡^	11.0 (83.0)^‡^	[50]
Taiwan (2001-2002)	Pregnant women	31–39	100	— (—)	52.4 (928.0)^‡^	10.3 (269.0)^‡^	1.2 (55.0)^‡^	10.5 (218.0)^‡^	21.7 (617.0)^‡^	20.8 (645.0)^‡^	[45]
The Netherlands (2002–2006)	Pregnant women	18–41	99	117.0 (1150.0)	42.8 (197.0)	42.1 (249.0)	7.5 (95.8)	6.9 (82.8)	14.0 (86.2)	14.5 (104.0)	[51]
Peru (2004)	Pregnant women	14–46	79	32.2^¶^ (—)	9.3^¶^ (—)	1.2^¶^ (—)	1.1^¶^ (—)	1.6^¶^ (—)	4.1^¶^ (—)	3.1^¶^ (—)	[52]
Spain (2004–2008)	Pregnant women	17–43	120	755.0 (—)	27.5 (—)	29.9 (—)	10.5 (—)	4.4 (—)	17.3 (—)	15.7 (—)	[37]
Germany (2005)^b^	Males and females	14–60	399	— (—)	49.6 (171.5)	44.9 (182.6)	7.2 (45.6)	4.9 (21.7)	19.2 (21.7)	14.7 (56.0)	[53]
Japan (2005–2008)	Pregnant women	31.9^¥^	149	6.0 (1067.0)^‡^	48.1 (504.0)^‡^	— (—)	3.5 (992.0)^‡^	4.4 (70.3)^‡^	8.6 (89.7)^‡^	9.2 (132.0)^‡^	[54]
Israel (2011)	Males and females	20–74	248	— (—)	27.9 (90.8)^*◊*^	37.6 (89.0)^*◊*^	4.3 (20.5)^*◊*^	11.2 (49.3)^*◊*^	30.4 (91.1)^*◊*^	17.1 (55.5)^*◊*^	[55]
Mexico 2007	Females	32–79	108	83.2^¶^ (—)	72.4^¶^ (—)	8.4^¶^ (—)	4.4^¶^ (—)	5.2^¶^ (—)	45.8^¶^ (—)	31.8^¶^ (—)	[56]
France 2007	Pregnant women	—	279	43.5 (600.7)	35.7 (201.1)	53.7 (274.1)	10.1 (88.7)	16.7 (266.6)	41.9 (605.1)	28.5 (427.9)	[57]
Denmark (2007–2009)	Males	19.5^¥^	881	78.0 (1936.0)	28.0 (91.0)	58.0 (173.0)	34.0 (164.0)	4.0 (18.0)	23.0 (79.0)	14.0 (55.0)	[58]
Canada (2007–2009)	Males and females	6–49	3236	49.1 (824.2)	23.8 (120.9)	— (—)	12.3 (81.9)	3.5 (24.9)	23.4 (180.3)	14.0 (113.8)	[47]
USA (2009-2010)	Males and females	≥6	2749	54.9 (988.0)	15.9 (75.9)	8.3 (41.3)	6.7 (48.3)	1.5 (14.1)	12.9 (103.0)	8.0 (55.7)	[38]
China (2010)	Males and females	10–40	183	21.5 (1330.0)^‡^	61.2 (798.0)^‡^	56.7 (791.0)^‡^	0.6 (43.0)^‡^	2.1 (207.0)^‡^	11.3 (1120.0)^‡^	7.0 (564.0)^‡^	[59]
Korea (2011)^c^	Males, females, and mothers	20–39	562	— (—)	— (—)	— (—)	— (—)	9.5 (94.0)	27.6 (98.2)	21.1 (82.3)	[48]
Denmark (2011)	Mothers	31–52	145	29.0 (359.0)	20.0 (70.0)	36.0 (139.0)	4.0 (22.0)	1.7 (6.9)	12.0 (50.0)	6.1 (21.0)	[39]
Belgium (2011-2012)	Mothers	≤45	125	34.0 (240.0)	31.0 (119.0)	33.0 (175.0)	6.4 (23.0)	2.3 (9.1)	11.0 (51.0)	7.6 (13.0)	[49]
Italy (—)^d^	Males and females	19–58	157	59.0 (748.6)	24.2 (143.0)	— (—)	16.7 (102.9)	3.1 (13.4)	12.1 (49.4)	— (—)	[60]

*N*: number of participants.

^‡^maximum.

^¥^arithmetic mean.

^¶^geometric mean.

—: no results/information.

^*◊*^Percentile 90th.

^a^Arithmetic mean of 2-3 and 5-6 years groups medians.

^b^Urine collected over eight consecutive days for each participant except one for seven days.

^c^12 h urine. Arithmetic mean of male, female, and mother medians.

^d^Arithmetic mean of female and male medians.
